# Citrus Peel Flours: From Residues to Bioactive Ingredients for Food Applications

**DOI:** 10.3390/molecules31101673

**Published:** 2026-05-15

**Authors:** Daniele Sales, Tiane C. Finimundy, Jessica Ribeiro, Sandrina Heleno, Filipa Mandim, Marina Kostić, Marina Soković, Lillian Barros, Cristina Caleja, Eliana Pereira

**Affiliations:** 1 CIMO, LA SusTEC, Instituto Politécnico de Bragança, Campus de Santa Apolónia, 5300-253 Bragança, Portugaltiane@ipb.pt (T.C.F.); jessica.ribeiro@ipb.pt (J.R.); sheleno@ipb.pt (S.H.); filipamandim@ipb.pt (F.M.); lillian@ipb.pt (L.B.); eliana@ipb.pt (E.P.); 2 Microbiology and Antibiotic Resistance Team (MicroART), Department of Veterinary Sciences, University of Trás-os-Montes and Alto Douro, 5000-801 Vila Real, Portugal; 3 Associated Laboratory for Green Chemistry (LAQV-REQUIMTE), Department of Chemistry, University NOVA of Lisbon, 2829-516 Lisbon, Portugal; 4 Institute for Biological Research “Siniša Stanković”—National Institute of the Republic of Serbia, University of Belgrade, Bulevar despota Stefana 142, 11108 Belgrade, Serbia; marina.kostic@ibiss.bg.ac.rs (M.K.); mris@ibiss.bg.ac.rs (M.S.); 5 Department of Pathologic Physiology, First Moscow State Medical University I.M. Sechenov (Sechenov University), Trubetskaya Street, House 8, Building 2, 119991 Moscow, Russia

**Keywords:** citrus residues, bioactivity, functional ingredients, circular economy, food applications

## Abstract

The high consumption of citrus fruits generates large amounts of peel bioresidues, whose valorization represents an important strategy for sustainable agri-food systems. This study aimed to characterize the nutritional, chemical, and bioactive properties of flours obtained from orange (FL), tangerine (FT), lime (FLA), and lemon (FLO) peels, and to evaluate their potential as functional food ingredients. The flours were evaluated for proximate composition, organic acids, phenolic compounds, fatty acids, free sugars, and bioactive properties. Lime flour showed the highest protein, ash, dietary fiber, and total phenolic contents, with hesperidin identified as the predominant compound. The corresponding extracts exhibited relevant antioxidant, antimicrobial, antiproliferative, and nitric oxide (NO) production inhibitory activities, with lime flour presenting the strongest overall bioactive potential. Based on these results, lime flour was selected for application in a food model by partially replacing wheat flour (10% and 20%) in “Madalenas”, a traditional Portuguese muffin cake. The incorporation of lime flour improved product preservation compared with the control formulation and samples containing a synthetic preservative (potassium sorbate). These findings highlight the potential of citrus peel flours, particularly lime flour, as natural functional ingredients and sustainable alternatives for food formulations, contributing to waste valorization and circular economy approaches in the agri-food sector.

## 1. Introduction

Citrus fruits represent a diverse group of economically important crops widely cultivated and consumed worldwide [1]. They originated in Southeast Asia, particularly in regions corresponding to present-day China and India, and were subsequently disseminated to the Mediterranean basin through ancient trade routes. From there, citrus cultivation expanded to the Americas, where it remains of significant economic importance [2]. According to recent FAO data, China remains the world’s leading producer of citrus fruits, followed by Brazil, both maintaining consistently high production levels in recent years [1]. In Europe, Spain continues to be the main citrus producer, with annual production of several million tons, while Portugal has a comparatively smaller production due to its territorial size; nevertheless, citrus fruits remain among the most important fruit crops at the national level, alongside pome fruits and vineyards [3].

According to data provided by the FAO regarding global citrus fruit production, the most representative species are oranges, tangerines, lemons, and limes, accounting for most of the production worldwide [1]. Citrus fruits possess a complex composition of biochemical compounds that contribute to their color, flavor, nutritional quality, and functional properties. Citrus peels are rich in dietary fiber, phenolic compounds, flavonoids, essential oils, and other phytochemicals with recognized antioxidant and health-promoting effects [4]. The high consumption of these fruits is linked to their attractive sensory characteristics and to increasing consumer interest in health and wellness, given the well-documented nutritional value and potential health benefits associated with citrus bioactives and juices [5].

However, the large production and consumption of these fruits generate significant amounts of by-products, raising concerning economic and environmental issues. In the fruit and vegetable industry, large quantities of fruit are discarded because they do not meet commercial quality standards, and substantial bio-waste is generated during different stages of industrial processing, leading to environmental pollution and economic losses [6]. Peels, seeds, and unused pulp are among the by-products frequently discarded as waste. Although several studies have reported the presence of bioactive compounds in citrus peels [7,8,9], these by-products are particularly rich in phenolic compounds, especially flavonoids such as hesperidin, naringin, and polymethoxyflavones, as well as carotenoids, limonoids, and essential oils, which have been associated with antioxidant, antimicrobial, anti-inflammatory, and antiproliferative activities. These compounds have been widely reported to exhibit strong antioxidant capacity and inhibitory effects against foodborne microorganisms, supporting their potential application as functional food ingredients [7,8,9]. However, their use in the form of flours and their direct incorporation into bakery products remain relatively underexplored, particularly regarding combined nutritional, chemical, and bioactive characterization.

Given this current problem, the valorization of citrus processing by-products has emerged as a sustainable strategy within the circular economy framework, aiming to convert these residues into value-added food ingredients. In this context, the present study aimed to develop flours from citrus peels (orange, tangerine, lemon, and lime), characterize their nutritional and chemical composition, and evaluate their bioactive properties. Furthermore, the most promising flour was selected for application as a partial wheat flour substitute in a bakery product (“Madalenas”) to assess its potential as a functional ingredient and its contribution to product preservation. This approach provides new insights into the valorization of citrus processing by-products and highlights their potential as sustainable sources of natural bioactive ingredients for food applications.

## 2. Results and Discussion

### 2.1. Nutritional Composition of Citrus Peel Flours

The nutritional composition of citrus peel flours is presented in Table 1. Significant differences (*p* < 0.05) were observed among the samples for all evaluated parameters, indicating species-dependent variability in peel composition.

Lime flour (FLA) exhibited the highest ash content (1.18 g/100 g), indicating a greater mineral concentration compared with the other flours. This sample also presented the highest protein (1.85 g/100 g) and dietary fibre contents (15.6 g/100 g), reflecting a comparatively higher nutritional density. In contrast, lemon flour (FLO) presented the lowest values for protein, carbohydrates, fibre, and energy, resulting in the lowest caloric value (69.2 kcal/100 g) among the samples.

Carbohydrate contents were similar in orange (FL) and tangerine (FT) flours (≈25.5 g/100 g), while significantly lower values were observed for lime and lemon flours, particularly for FLO. Fat content was generally low in all samples, although lemon flour presented significantly lower values than the remaining flours. Energy values reflected the differences observed in carbohydrate and fibre contents, with FL and FT showing the highest caloric values and FLO the lowest.

The nutritional profiles align with previous reports on citrus peels, which describe these by-products as rich sources of dietary fibre, minerals, and structural carbohydrates [10]. Compared to the edible portions of fresh citrus fruits, citrus peels present a higher nutritional density, particularly regarding dietary fibre and protein content. Similar trends have been consistently reported for fruit by-products, reinforcing their relevance as alternative food matrices with enhanced nutritional value [11]. Compared to previous studies, which often focus on a single citrus species or only on peel characterization, this work provides a comprehensive comparison of four citrus flours and their nutritional and functional potential.

Overall, the results indicate that citrus peel flours, particularly lime flour, are valuable nutrient sources with functional potential. Their high fibre and protein contents, low fat levels, and mineral richness support their incorporation into food formulations, aligning with sustainable food system strategies and circular economy principles. These findings underscore the importance of integrating citrus by-products into food applications, contributing to waste reduction, resource efficiency, and the development of health-promoting, sustainable food ingredients.

### 2.2. Chemical Composition of Citrus Flours

The free sugar composition of the citrus peel flours is presented in Table 2. Fructose, glucose, sucrose, and trehalose were detected in all samples, with statistically significant differences (*p* < 0.05) among citrus species.

FL exhibited the highest concentrations of fructose and glucose (3.9 g/100 g each), confirming the predominance of monosaccharides in this matrix. FT presented a distinct profile, with sucrose as the major sugar (2.9 g/100 g), followed by fructose and glucose. In contrast, FLA and FLO flours showed significantly lower total free sugar contents, with glucose being the dominant monosaccharide in both samples.

Recent studies have consistently reported fructose and glucose as the main free sugars in citrus-derived matrices, including peels and pomace, although their relative abundance varies according to species, cultivar, and processing conditions [12]. The lower sugar levels observed in lime and lemon flours agree with current literature, which describes these species as having a higher proportion of organic acids and structural polysaccharides in the peel, resulting in reduced accumulation of free sugars [13].

Sucrose predominance in tangerine peel flour has also been reported in recent compositional studies on citrus by-products, where mandarins and related species tend to retain higher sucrose contents compared to lemons and limes [10]. These differences are often associated with varietal characteristics and metabolic partitioning during fruit maturation and post-harvest processing.

Trehalose was detected at low concentrations in all samples (0.17–0.30 g/100 g). Although present in minor amounts, its detection is noteworthy, as trehalose has only recently been quantified in citrus by-products using high-resolution chromatographic techniques. Recent evidence suggests that trehalose may be associated with stress-related metabolic responses in plant tissues, including dehydration and thermal processing, which could explain its presence in dried citrus peel flours [14].

From a nutritional and technological perspective, the relatively low free sugar content of citrus peel flours, particularly in lime and lemon samples, represents a significant advantage for their incorporation into food formulations. Recent studies emphasize that fruit by-products with reduced free sugar levels and high fibre content are especially suitable for the development of functional bakery products and clean-label formulations, contributing to lower glycemic impact and improved shelf stability [7].

The individual fatty acid profile and the relative proportions of saturated (SFA), monounsaturated (MUFA), and polyunsaturated fatty acids (PUFA) of citrus peel flours (FL, FT, FLA, and FLO) are presented in Table 2. Fatty acids are essential dietary components, and their qualitative distribution is considered more relevant than total lipid content when evaluating the nutritional quality of plant-derived matrices.

A total of 19 fatty acids were identified across the samples, revealing marked qualitative and quantitative differences among citrus species. In all flours, linoleic acid (C18:2n6c) was the predominant fatty acid, accounting for 32.4–50.5% of total fatty acids, followed by α-linolenic acid (C18:3n3), oleic acid (C18:1n9c), and palmitic acid (C16:0). This distribution is consistent with recent reports describing citrus peels as lipid matrices enriched in essential polyunsaturated fatty acids [8,13].

Polyunsaturated fatty acids constituted the major lipid fraction in all samples, ranging from 49.5 ± 0.3% in FLO to 64.3 ± 0.1% in FLA. The significantly higher PUFA content observed in FLA is mainly attributed to its elevated levels of α-linolenic acid (C18:3n3), which reached approximately 17% of total fatty acids. This result highlights lime peel flour as a particularly rich source of ω-3 fatty acids, which are widely recognized for their role in modulating inflammatory processes, improving endothelial function, and reducing cardiovascular risk [9].

Linoleic acid (C18:2n6c), the main ω-6 fatty acid, was present at high levels in all samples, with the highest proportion detected in FT (50.5 ± 0.1%). Adequate dietary intake of linoleic acid is essential, as it serves as a precursor for bioactive lipid mediators and contributes to the maintenance of membrane integrity [15]. The simultaneous presence of both linoleic and α-linolenic acids results in a nutritionally favourable PUFA profile, particularly in FL, FT, and FLA samples.

Saturated fatty acids represented the second most abundant fraction. FLO exhibited a significantly higher SFA content (41.8 ± 0.3%) compared to the remaining flours, which showed similar values (approximately 22–24%). This increase is mainly associated with higher concentrations of palmitic acid (C16:0) and long-chain saturated fatty acids such as behenic (C22:0), tricosanoic (C23:0), and lignoceric acids (C24:0), which were detected almost exclusively in FLO. These findings suggest species-specific differences in lipid metabolism and structural lipid composition of citrus peels, as also reported in recent comparative studies [13].

Monounsaturated fatty acids were present in lower proportions, with oleic acid (C18:1n9c) as the dominant MUFA in all samples. FL and FT flours exhibited the highest MUFA contents (16.8 ± 0.2% and 16.1 ± 0.1%, respectively), whereas FLO showed significantly lower levels (8.72 ± 0.03%). Despite their lower abundance, MUFAs are considered nutritionally relevant due to their documented role in improving lipid profiles and reducing low-density lipoprotein cholesterol without adversely affecting high-density lipoprotein levels [9].

Overall, the citrus peel flours, particularly FL, FT, and FLA, displayed a favourable PUFA/SFA ratio, which is considered an important indicator of lipid quality from a nutritional perspective. The lipid profile observed, combined with the low total fat content of these flours, reinforces their potential application as functional food ingredients capable of improving the nutritional lipid profile of formulated products. These results further support the valorization of citrus peel residues within a circular economy framework, aligning sustainability with nutritional and technological benefits [7].

The organic acid composition of citrus flours is presented in Table 2. Six organic acids were identified across the samples, namely oxalic, quinic, malic, citric, fumaric, and ascorbic acids. Organic acids are key constituents of citrus matrices and play an important role in nutritional quality, bioactivity, and technological performance.

FT exhibited the highest total organic acid content (5.07 ± 0.02 g/100 g), followed by FL, FLA, and FLO. This variability reflects species-dependent metabolic pathways and acid accumulation patterns, which have been widely reported for citrus fruits and their by-products [7,8].

Citric acid was the predominant organic acid in FL and FT, accounting for the major contribution to total organic acids, particularly in FT (4.63 ± 0.01 g/100 g). The dominance of citric acid in sweet citrus varieties is well documented and is closely associated with acidity, flavour balance, and preservative capacity of citrus-derived ingredients [13]. In contrast, citric acid was not detected in FLA and FLO, which exhibited a distinct organic acid profile.

Quinic acid was the major organic acid in FLA and FLO, reaching 0.99 ± 0.04 g/100 g and 0.62 ± 0.02 g/100 g, respectively, and was not detected in FL and FT. This finding is consistent with recent studies reporting higher quinic acid levels in more acidic citrus species, particularly lime and lemon, and highlights clear species-specific differences in organic acid biosynthesis [7].

Oxalic acid was detected in all samples, with values ranging from 0.106 ± 0.001 g/100 g in FLO to 0.15 ± 0.01 g/100 g in FLA. Although present at lower concentrations, oxalic acid contributes to overall acidity and has been reported as a common constituent of citrus peels [8]. Malic acid was detected in FT, FLA, and FLO, with the highest concentration observed in lemon flour (0.20 ± 0.01 g/100 g), whereas it was not detected in FL.

Fumaric acid was present in trace amounts in FT, FLA, and FLO, reflecting its minor contribution to the total organic acid pool. Despite its low concentration, fumaric acid is recognized for its antimicrobial properties and technological relevance in food systems [16].

Ascorbic acid was detected in all citrus peel flours, with FLO exhibiting the highest concentration (0.030 ± 0.001 g/100 g). The presence of ascorbic acid after drying and milling processes highlights the potential of citrus peels as a source of natural antioxidants. Recent studies have demonstrated that citrus by-products retain appreciable amounts of ascorbic acid, contributing synergistically with phenolic compounds to overall antioxidant capacity [17].

Overall, the organic acid profiles of citrus peel flours are strongly dependent on citrus species, with distinct dominance patterns of citric and quinic acids. These compositional differences may influence not only bioactivity but also the sensory and preservative properties of food formulations incorporating these flours. The results reinforce the potential of citrus peel flours as multifunctional ingredients, supporting their valorisation within a circular economy framework.

The phenolic composition of the hydroethanolic extracts obtained from citrus peel flours is presented in Table 3. A total of forty-six phenolic compounds were identified, including twenty-nine flavonoids and seventeen phenolic acids and their derivatives. Citrus peels are widely recognized as one of the richest sources of phenolic compounds among fruit by-products, and the diversity observed in this study reflects both species-specific metabolic pathways and the efficiency of hydroethanolic extraction.

Marked differences were observed among the citrus species in terms of total phenolic content and qualitative composition. FLA exhibited the highest total phenolic content, with a clear predominance of flavonoids. Hesperidin was the major compound detected (23.5 ± 0.2 mg/g extract), followed by limocitrol-*O*-hexoside-*O*-deoxyhexoside (13.2 ± 0.2 mg/g), limocitrin-glucosyl-3-hydroxy-3-methylglutaroyl-glucoside (6.4 ± 0.2 mg/g), and apigenin-6,8-C-diglucoside (5.0 ± 0.2 mg/g). These compounds are characteristic flavonoids of citrus matrices and have been widely associated with antioxidant, anti-inflammatory, and antimicrobial activities [13].

FL presented the second highest total phenolic content (27.0 ± 0.4 mg/g extract), followed by FLO (17.3 ± 0.3 mg/g) and FT (13.5 ± 0.3 mg/g). In these three flours, phenolic acids and their derivatives were the dominant compounds. Among them, 3-*O*-caffeoylquinic acid and galloyl-deoxyhexoside were the most abundant, with concentrations ranging from 1.93 to 3.18 mg/g extract, depending on the citrus species. Phenolic acids are known to contribute significantly to antioxidant capacity and may act synergistically with flavonoids to enhance overall bioactivity [8].

The predominance of hesperidin in lime peel flour is particularly relevant, as this flavanone has been extensively studied for its biological properties, including radical scavenging activity, modulation of inflammatory pathways, and antimicrobial effects. Recent studies have highlighted hesperidin-rich citrus by-products as promising ingredients for functional food development and natural preservation strategies [7].

Overall, the high number and diversity of phenolic compounds identified in citrus peel flours corroborate recent findings reporting citrus residues as phenolic-rich matrices with strong bioactive potential [13]. The differences observed among species further emphasize the importance of citrus variety selection when targeting specific functional properties. In this context, the elevated phenolic content of lime peel flour is consistent with its superior bioactivity observed in subsequent antioxidant and antimicrobial assays, reinforcing its suitability for application as a natural functional ingredient in food formulations.

Collectively, these chemical profiles demonstrate that citrus peel flours are multifunctional ingredients. Differences in sugars, lipids, organic acids, and phenolics among species provide opportunities to tailor formulations for nutritional, sensory, and functional purposes, supporting both valorisation of agro-industrial residues and the development of sustainable, circular food products.

### 2.3. Bioactive Potential

Antioxidant activity: The antioxidant activity of the hydroethanolic extracts obtained from citrus peel flours was evaluated using TBARS, DPPH radical scavenging and reducing power assays, with results expressed as EC_50_ values (Table 4).

Regarding lipid peroxidation inhibition (TBARS assay), significant differences were observed among samples. FLA extract exhibited the strongest activity, with an EC_50_ value of 0.29 ± 0.04 μg/mL, significantly lower than those obtained for FL, FT and FLO (*p* < 0.05). This result highlights the superior ability of FLA to prevent oxidative degradation of lipids, a key mechanism in food deterioration and biological oxidative stress.

A similar trend was observed in the DPPH radical scavenging assay. The FLA and FLO extracts showed significantly lower EC_50_ values (2.01 ± 0.03 and 2.0 ± 0.3 μg/mL, respectively) compared to FL and FT, indicating a higher capacity to neutralize free radicals. These results suggest that lime and lemon peel flours contain flavonoid-rich compounds capable of effective hydrogen-donation, particularly hesperidin, limocitrin derivatives, and apigenin-6,8-C-diglucoside.

In contrast, the reducing power assay revealed a different antioxidant behavior. The FL extract demonstrated the highest reducing capacity, presenting the lowest EC_50_ value (0.89 ± 0.04 μg/mL), while FLO showed the weakest activity (4.3 ± 0.1 μg/mL). This assay-dependent variation reflects the influence of phenolic composition on specific antioxidant mechanisms, with flavonoids predominantly contributing to radical scavenging and lipid peroxidation inhibition, while phenolic acids such as 3-*O*-caffeoylquinic acid and galloyl-deoxyhexoside enhance electron-transfer-based reducing power.

The superior antioxidant performance of FLA in TBARS and DPPH assays is consistent with its higher concentration of total phenolic compounds and the predominance of flavonoids such as hesperidin, limocitrin and apigenin derivatives. These compounds are widely recognized for their ability to inhibit lipid peroxidation and scavenge reactive oxygen species through multiple mechanisms [13]. Conversely, the strong reducing power observed in FL may be attributed to the higher relative abundance of phenolic acids, including caffeoylquinic acid derivatives, which are particularly effective in electron transfer reactions [16].

Recent studies have consistently reported that citrus peel extracts exhibit stronger antioxidant activity than the edible pulp, highlighting their potential as natural antioxidants in food and nutraceutical applications [8]. The present results further confirm that citrus peel flours, especially lime peel flour (FLA), represent a valuable source of bioactive compounds with multifunctional antioxidant properties.

These findings indicate that specific phenolic compounds, rather than total phenolic content alone, are key contributors to the observed antioxidant effects. Overall, the pronounced antioxidant activity observed supports the valorisation of citrus peel by-products as functional food ingredients and natural alternatives to synthetic antioxidants, contributing to sustainable food processing and clean-label product development.

Antimicrobial activity: The antibacterial activity of the hydroethanolic extracts obtained from citrus peel flours was evaluated against a panel of foodborne microorganisms, including Gram-positive bacteria (*Staphylococcus aureus*, *Bacillus cereus*, and *Listeria monocytogenes*) and Gram-negative bacteria (*Escherichia coli*, *Salmonella typhimurium*, and *Enterobacter cloacae*). The results, expressed as minimum inhibitory concentration (MIC) and minimum bactericidal concentration (MBC), are presented in Table 4.

Overall, Gram-positive bacteria exhibited higher susceptibility to the extracts, with Bacillus cereus showing the lowest MIC and MBC values across all samples. This trend is consistent with recent studies reporting greater sensitivity of Gram-positive bacteria to plant-derived bioactive compounds, largely due to the absence of an outer membrane and higher membrane permeability [18].

When compared with the synthetic food preservatives used as controls (E211 and E224), the citrus peel extracts demonstrated comparable or superior inhibitory activity. Notably, all extracts showed lower MIC values against *Staphylococcus aureus* than E211, and consistently outperformed E224 against Bacillus cereus. In contrast, Escherichia coli was the most resistant microorganism, presenting higher MIC and MBC values relative to the controls, a behavior commonly associated with the structural complexity of Gram-negative bacterial membranes [19].

Regarding antifungal activity, all extracts exhibited inhibitory effects against the six tested fungal strains (Table 4). *Aspergillus fumigatus* was the most sensitive species, with MIC values lower than those of the reference preservatives for all extracts. For *Aspergillus niger*, the FL and FLO extracts showed stronger fungicidal activity than the controls, while FT and FLA exhibited comparable effects. For the remaining fungi, the fungicidal activity of the extracts was like that of E211, confirming their broad-spectrum antifungal potential.

The antimicrobial activity of the extracts can be attributed to the combined presence of flavonoids (e.g., hesperidin, limocitrin derivatives, apigenin glycosides) and phenolic acids, which act synergistically by disrupting microbial membranes, lowering intracellular pH, and interfering with essential metabolic pathways [8]. These results demonstrate a clear link between the phenolic composition of citrus peel flours and their broad-spectrum antimicrobial activity, supporting further exploration of specific compounds responsible for the observed effects.

Antiproliferative and NO-production inhibition: The antiproliferative activity of hydroethanolic extracts from citrus peel flours was evaluated against four human tumor cell lines (MCF-7, NCI-H460, AGS, and Caco-2), as well as against a primary non-tumor liver cell culture (PLP2). NO production inhibition was assessed using LPS-stimulated RAW 264.7 macrophages (Table 4).

Extracts obtained from FL, FT, and FLO did not exhibit antiproliferative capacity toward any of the tested tumor cell lines (GI_50_ > 400 μg/mL). A similar absence of toxicity was observed in PLP2 non-tumor cells, indicating a favourable safety profile. Comparable findings have been reported for citrus peel extracts rich in flavanone glycosides, which often show low cytotoxicity in vitro at moderate concentrations [20].

In contrast, FLA extract exhibited selective antiproliferative activity. Proliferation inhibition was observed in Caco-2, MCF-7, and NCI-H460 cells, with GI_50_ values of 140, 343, and 208 μg/mL, respectively, while no effect was detected against AGS cells (GI_50_ > 400 μg/mL). Importantly, FLA showed low cytotoxicity toward non-tumor PLP2 cells (GI_50_ = 385 μg/mL), suggesting selective activity against cancer cells. Similar selective antiproliferative effects have been reported for lime and lemon peel extracts, which are associated with high levels of hesperidin, apigenin derivatives, and C-glycosyl flavones [21].

The antiproliferative behaviour of FLA may be partially explained by its higher phenolic content and the predominance of flavonoids such as hesperidin and apigenin derivatives, compounds previously reported to modulate cell cycle progression and induce apoptosis in cancer cells [22].

Regarding NO-production inhibition, none of the studied extracts exhibit capacity to inhibit nitric oxide production in LPS-stimulated RAW 264.7 macrophages (GI_50_ > 400 μg/mL). Some studies in the literature report moderate anti-inflammatory effects of citrus peel through the negative regulation of pro-inflammatory mediators and oxidative stress pathways [23].

These results demonstrate a clear relationship between phenolic composition and selective antiproliferative activity, highlighting lime peel flour (FLA) as the most bioactive sample. The findings underscore species-specific differences in bioactivity and the role of key flavonoids, providing mechanistic insight into their contribution to antioxidant, antimicrobial, and antiproliferative effects.

### 2.4. Evaluation of the Effects of Incorporating Lime Flour into a Confectionery Product

Following the nutritional, chemical, and bioactive characterization of the citrus peel flours, FLA was selected for incorporation into a bakery product due to its superior antioxidant and antimicrobial properties. The aim was to evaluate its potential as a natural functional ingredient in “madalenas”, a traditional Portuguese sponge cake.

Four formulations were prepared: (i) control *madalenas* with wheat flour (MFT); (ii) *madalenas* with wheat flour and potassium sorbate as a synthetic preservative (MCS); (iii) *madalenas* in which 10% of the wheat flour was replaced by lime peel flour (M10); and (iv) *madalenas* with 20% wheat flour replacement (M20). The selected substitution levels of 10–20% citrus peel flour were based on previous studies reporting acceptable technological and sensory properties in bakery products [7]. Total replacement of wheat flour with lime peel flour was not feasible due to poor batter consistency. Potassium sorbate (E202) was used as a reference synthetic preservative at the maximum level permitted by European legislation (2000 mg/kg; Commission Regulation (EU) No 1129/2011), allowing a direct comparison of natural versus synthetic preservation effects.

Although no formal sensory evaluation was conducted, the use of citrus flours in bakery products is common in the industry and generally well-accepted by consumers. “Madalenas” were evaluated at 0, 3, and 6 days of storage for water activity, texture, colour, and microbial load. Data were analyzed by two-way ANOVA to assess the effects of storage time (ST), formulation (F), and their interaction (ST × F). When no significant interaction was observed (*p* > 0.05), post hoc multiple comparison tests were applied to evaluate the main effects of each factor. In cases where the interaction was significant (*p* < 0.05), the combined effect of storage time and formulation was interpreted using estimated marginal means to understand trends and relative contributions of each factor.

Table 5 presents the effects of lime peel flour incorporation and storage time on colour (CIE L*, a*, b*), water activity (aw), and texture of “madalenas”. Overall, the interaction between storage time and incorporation was not significant, except for the external a* coordinate (*p* < 0.05). External lightness (L*) increased slightly from day 0 to day 3, with no further change at day 6. Samples with 20% lime flour (M20) were darker (lower L*), while the control (MFT) and synthetic preservative (MCS) samples were the lightest. The b* coordinate (blue-yellow) was mainly affected by incorporation: 20% lime flour shifted slightly toward lower b* (less yellow), whereas the control tended toward higher b* (more yellow). For internal colour, L* and a* showed only small, no relevant changes over time, with only minor differences between control and citrus flour, incorporated samples that were visually negligible. Similarly, internal b* reflected a slight difference between control/synthetic preservative and citrus flour samples, with no temporal effect. These subtle changes can be mechanistically linked to the compositional and bioactive profile of lime peel flour, including higher dietary fiber and phenolic content, which contributed to minor increases in hardness and chewiness and to improved product preservation through natural antioxidant and antimicrobial effects.

Table 5 presents water activity (aw) and texture parameters of “madalenas” across storage times and formulations. Storage time significantly affected aw (*p* < 0.05), which showed a slight decrease by day 6, likely contributing to increased hardness due to dehydration. Control samples (100% wheat flour) were less hard than samples with lime flour, likely reflecting the lower gluten content in citrus flour, as gluten contributes to dough viscoelasticity [24]. Lower aw is beneficial for product preservation, as microbial growth is limited [25]. Elasticity was not significantly affected by either storage or incorporation (*p* > 0.05). In contrast, cohesiveness, viscosity, chewiness, and resilience were influenced by the interaction of time and incorporation. Increased chewiness over time and with lime flour addition corresponds to higher hardness and reduced cohesiveness, consistent with established texture relationships [26]. Although minor textural differences exist between control and lime flour, incorporated “madalenas”, these are unlikely to impair sensory acceptability. Overall, these results demonstrate that lime peel flour can be incorporated as a functional ingredient in bakery products, enhancing preservation and supporting the valorization of citrus by-products.

#### Microbial Load Assessment

**Figure 1:**
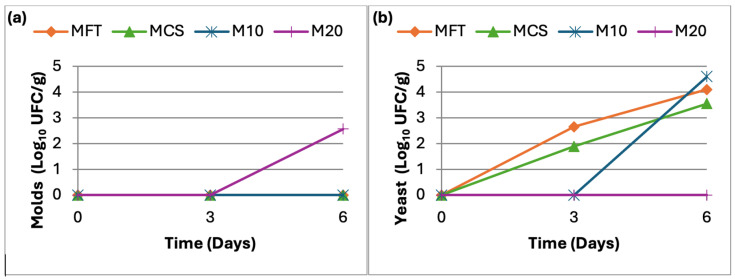
(**a**) Mold counts over a 6-day period; (**b**) Yeast counts over a 6-day period.

Microbial load was evaluated for each “madalenas” formulation on the day of preparation (T0), and after 3 (T3) and 6 days (T6) of storage. During this period, samples were kept at room temperature in sealed plastic bags. The evolution of molds and yeasts over the storage time is presented in Figure 1. Mold growth was detected only in the control sample produced with wheat flour and without either synthetic preservative or lime flour (MFT). No mold development was observed in the remaining formulations throughout the 6-day storage period. Regarding yeasts, MFT and the sample containing the synthetic preservative (MCS) showed growth after 3 days of storage, whereas the formulation incorporating 10% lime flour (M10) exhibited yeast growth only after 6 days. No yeast growth was detected in the formulation containing 20% lime flour (M20).

**Figure 2:**
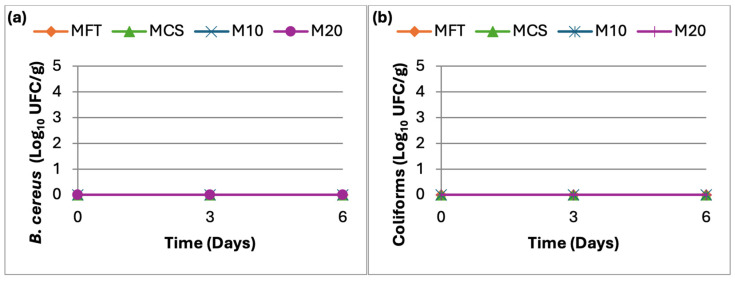
(**a**) *B. cereus* counts over a 6-day period; (**b**) Coliform counts over a 6-day period.

The enumeration of *B. cereus* and total coliforms (Figure 2) revealed that these microorganisms were not detected in any formulation during the entire storage period, indicating satisfactory hygienic conditions and absence of post-processing contamination.

**Figure 3:**
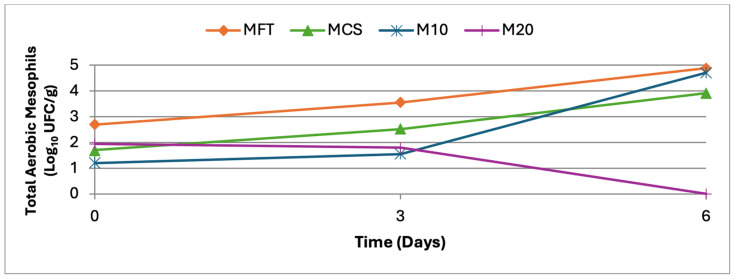
Total aerobic mesophilic counts over a 6-day period.

Total aerobic mesophilic counts (Figure 3) were detected in all samples from the day of preparation onward. A gradual increase was observed in MFT, MCS, and M10 during storage, while M20 showed a slight decrease at day 6, although without statistically significant differences (*p* > 0.05).

Altogether, the microbial assessment suggests that lime flour exerted a stronger inhibitory effect on microbial growth than the commonly used synthetic preservative. This finding is particularly relevant, as the incorporation of citrus-derived flour not only enhances the nutritional profile and provides bioactive compounds with potential health benefits, but also contributes to improved microbiological stability and extended shelf life of the product.

These results indicate that the antimicrobial effect of lime peel flour in “madalenas” is likely linked to its rich content of flavonoids, organic acids, and phenolic compounds, which may act synergistically to inhibit microbial growth through disruption of cell membranes and reduction in intracellular pH. The differential response among formulations highlights the importance of both incorporation level and the specific bioactive composition of the citrus peel flour. This mechanistic insight demonstrates that beyond simple preservation, lime peel flour can serve as a multifunctional ingredient, providing natural antimicrobial protection while enhancing the nutritional and functional quality of bakery products.

## 3. Materials and Methods

### 3.1. Sample Preparation

Fresh citrus fruits, *Citrus sinensis* L. Osbeck (orange), *Citrus reticulata* Blanco (tangerine), *Citrus latifolia* Tanaka (lime), and *Citrus limonum* (L.) Burm (lemon) were purchased from local market in the city of Bragança, Portugal. Fruits were visually inspected and selected for uniform appearance and maturity stage (ripe, free of visible defects), then were washed and manually peeled, and the peels were oven-dried at 40 °C for 3 days to ensure moisture removal while minimizing the degradation of thermolabile compounds. Dried peels were ground into a fine, homogeneous powder using an automatic grinder (Moulinex La moulinette AD560120, Moulinex, Alençon, France) and stored in a cool, dry place protected from light until further analysis.

### 3.2. Proximate Nutritional Composition

Proximate composition was determined separately for each citrus flour. For this purpose, the AOAC official analytical methodologies were used [27]. Moisture was determined gravimetrically by desiccation in an oven at 105 °C to constant weight, according to AOAC 984.25. Crude protein (N × 6.25) was determined by the macro-Kjeldahl method (AOAC 991.02). Fat content was determined by Soxhlet extraction with petroleum ether according to AOAC 989.05. The total dietary fiber content was determined using the AOAC 985.29 method with the Kit TDF100A (Sigma-Aldrich Chemie GmbH, Buchs, Switzerland). Total mineral content was determined as ash by incineration at 550 °C (AOAC 935.42). Results are expressed as g/100 g of fresh sample. Total carbohydrates were calculated by difference. Energy was calculated, and results were expressed in kcal/100 g.

### 3.3. Analysis of Soluble Sugars

Soluble sugars were determined following a previously described method [28]. Samples were extracted with ethanol/water (80:20, *v*/*v*) at 80 °C for 90 min, using melezitose (25 mg/mL) as an internal standard. Sugar analysis was performed by HPLC coupled to a refractive index detector, using a Eurospher 100-5 NH_2_ column (4.6 × 250 mm, 5 µm). Separation was achieved with acetonitrile/water (70:30, *v*/*v*) as the mobile phase. Sugars were identified by comparison with authentic standards and quantified relative to the internal standard. Results were expressed as g/100 g of fresh sample.

### 3.4. Analysis of Fatty Acids Profile

Fatty acid methyl esters (FAMEs) were prepared from the lipid extracts obtained during fat determination through acid-catalysed transesterification. Oils were reacted with a methanol-sulfuric acid-toluene solution (2:1:1, *v*/*v*/*v*) and incubated at 50 °C overnight under agitation. After phase separation with water and diethyl ether, the organic phase containing FAMEs was dried over anhydrous sodium sulfate, filtered (0.22 µm), diluted in diethyl ether, and stored at −20 °C until analysis.

FAMEs were analysed by gas chromatography with flame ionization detection (GC-FID) using a capillary column (30 m × 0.32 mm × 0.25 µm, Macherey-Nagel). Fatty acids were identified by comparison of retention times with a standard FAME mixture, following chromatographic conditions previously described by [29]. Results were expressed as relative percentages.

### 3.5. Analysis of Organic Acids

Organic acids were analyzed by ultra-fast liquid chromatography coupled to a diode array detector (UFLC-DAD, Shimadzu 20A series, Shimadzu, Kyoto, Japan), following a previously described method [29]. Samples were extracted with 4.5% metaphosphoric acid at room temperature and agitation, protected from light. After filtration, extracts were analyzed using a reverse-phase C18 column maintained at 35 °C, with sulfuric acid (3.6 mM) as the mobile phase. Detection was performed at 215 nm and 245 nm for ascorbic acid. Organic acids were identified and quantified using external calibration curves prepared from commercial standards. Results were expressed as g/100 g of fresh weight.

### 3.6. Extraction and Profiling of Phenolic Compounds and Their Bioactive Properties

Flour samples (2 g) were extracted twice with ethanol/water (80:20, *v*/*v*) under magnetic stirring (150 rpm) at room temperature for 1 h. After extraction, the mixtures were filtered through Whatman No. 1 filter paper to remove solid residues. The combined filtrates were concentrated under reduced pressure at 60 °C using a rotary evaporator (Heidolph, Schwabach, Germany) until approximately 80% of the initial volume was removed. The remaining aqueous phase was frozen, lyophilized (Labconco Freeze Zone 6, Kansas City, MO, USA), and the resulting dry extracts were used for the bioactivity assays described in Section 3.6.1 and Section 3.6.2.

#### 3.6.1. Phenolic Composition

Phenolic compounds were characterized using an HPLC-DAD-ESI/MS system (Dionex Ultimate 3000 UPLC, Thermo Scientific, San Jose, CA, USA) equipped with a quaternary pump, autosampler (kept at 5 °C), degasser, and thermostatted column compartment. Separation was achieved on a Waters Spherisorb S3 ODS-2 C18 column (4.6 × 150 mm, 3 µm) maintained at 35 °C. The mobile phase consisted of (A) 0.1% formic acid in water and (B) acetonitrile, with the following gradient: 10–15% B (0–5 min), 15–20% B (5–10 min), 20–25% B (10–20 min), 25–35% B (20–30 min), 35–50% B (30–40 min), followed by column re-equilibration for 10 min. The flow rate was 0.5 mL/min. Detection was carried out using a diode array detector (DAD) at 280, 330, and 370 nm, coupled to a mass spectrometer (LTQ XL Linear Ion Trap, ThermoFinnigan, San Jose, CA, USA) equipped with an electrospray ionization (ESI) source operating in negative mode (100–1500 *m*/*z*). Compound identification was performed by comparison of retention times, UV–Vis spectra, and mass spectral data with authentic standards when available; otherwise, compounds were tentatively identified based on literature data and MS fragmentation patterns. Quantification was performed using calibration curves of available standards or structurally related compounds. Results were expressed as mg/g of extract.

#### 3.6.2. Evaluation of Bioactive Potential

Antioxidant activity: To evaluate antioxidant activity, the extract obtained under optimized conditions was initially redissolved in water (2.5 mg/mL) and then successively diluted. Thiobarbituric acid reactive substances (TBARS), reducing power (RP), and 2,2-diphenyl-1-picrylhydrazyl (DPPH) radical-scavenging activities were measured as described by [30]. Trolox was used as the positive control. The results were expressed as IC_50_ (mg/mL), which corresponds to the extract concentration required to provide 50% of the antioxidant activity.

Antiproliferative activity: Antiproliferative activity was evaluated using sulphorodamine B colorimetric assay, according to the procedure described by [31]. The following tumor cell lines were used: AGS (gastric adenocarcinoma), Caco-2 (colon adenocarcinoma), MCF-7 (breast carcinoma), and NCI-H460 (lung carcinoma). Additionally, a non-tumoral pig liver cell line (PLP2), established as described previously [32], was tested. The studied extracts were dissolved in water to obtain a concentration of 8 mg/mL, which was further diluted to obtain a range of concentrations to be tested (final concentrations, between 400 and 6.25 μg/mL). Ellipticine (Sigma-Aldrich, St Louis, MO, USA) was used as the positive control, while cells without samples were used as the negative control. Results were expressed as GI_50_ (concentration of the extract required to inhibit 50% of cell proliferation, μg/mL).

Nitric oxide (NO)-production inhibition: The extract’s capacity to inhibit nitric oxide (NO) production, a chemical mediator of the inflammatory process, was evaluated through lipopolysaccharide (LPS)-stimulated murine macrophage cell line (RAW 264.7), according to the method described by [33]. The concentrations tested were obtained as described in the antiproliferative procedure. Dexamethasone (Sigma-Aldrich, Saint Louis, MO, USA) was used as a positive control, and cells with and without LPS as a negative control. The obtained results were expressed in IC_50_ values (μg/mL).

Antimicrobial activity: The antibacterial potential of the optimized extract was evaluated using a methodology described by [34]. Three Gram-positive bacteria, namely, *Staphylococcus aureus* (ATCC 11632), *Bacillus cereus* (food isolate), *Listeria monocytogenes* (NCTC 7973), and three Gram-negative bacteria, namely, *Escherichia coli* (ATCC 25922), *Salmonella enterica* serovar. Typhimurium (ATCC 13311) and *Enterobacter cloacae* (ATCC 35030) were tested. Additionally, the antifungal activity was assessed against six micromycetes: *Aspergillus fumigatus* (human isolate), *Aspergillus niger* (ATCC 6275), *Aspergillus versicolor* (ATCC 11730), *Penicillium funiculosum* (ATCC 36839), *Trichoderma viride* (IAM 5061) and *Penicillium verrucosum* var. *cyclopium* (food isolate), using the methodology described by [34]. The results were presented as minimum inhibitory concentration (MIC), minimum bactericidal concentration (MBC), and minimum fungicidal concentration (MFC) and were expressed in mg/mL. Broth was used as a negative control and commercial preservatives sodium sulfite (E221) and potassium metabisulfite (E224) were used as positive controls.

### 3.7. Development of a New Confectionery Product Using Improved-Performing Citrus Flour

Based on the nutritional, chemical, and bioactive characterization of the citrus flour, lime flour (FLA) was selected for application as a natural ingredient in a bakery product. The flour was incorporated as a partial substitute for wheat flour in “*madalenas*”, a traditional Portuguese muffin-style cake.

The traditional formulation consisted of: 100 g wheat flour, 50 g sugar, 50 mL milk, 30 g butter, 1 egg (≈50 g), and 2 g baking powder. Four formulations were prepared: (a) MFT (control): containing only wheat flour; (b) MCS: wheat flour with 0.1% potassium sorbate (E-202) as a synthetic preservative; (c) M10: 10% of wheat flour replaced by lime flour; (d) M20: 20% of wheat flour replaced by lime flour.

All products were prepared according to the traditional recipe, maintaining constant ingredient proportions, mixing conditions, and baking parameters. The batter was portioned into ≈40 g units, baked at 180 °C for 25 min, cooled to room temperature, and stored in sealed packaging under ambient conditions. Samples were evaluated immediately after production (T0) and after 3 (T3) and 6 (T6) days of storage.

#### 3.7.1. Evaluation of the Physical Properties of “Madalenas”

The surface colour of the “madalenas” was measured in triplicate by each group of samples, at three different points using a colorimeter (model CR-400, Konica Minolta Sensing Inc., Tokyo, Japan). Illuminant C and an 8 mm diaphragm aperture were used, after calibration on a white standard tile.

The texture of the “madalenas” was evaluated using a TA.XT texture analyzer (Stable Micro Systems, Surrey, UK) equipped with Exponent software (version 6.1.16.0) and a cylindrical probe (P/36R). The test conditions were a pre-test speed of 2 mm/s, a post-test speed of 3 mm/s, and a test speed of 3 mm/s, with a contact force of 10 g. The analysis was performed in triplicate for each batch of “madalenas” produced, following the procedure described by [26].

To evaluate the water activity of the “madalenas” an AquaLab 4TE water activity analyzer (Meter Group, Pullman, WA, USA) was used, and the analysis was performed in triplicate for each formulation and storage time.

#### 3.7.2. Microbial Load

For microbial load determination, ten grams of each sample were aseptically transferred into 90 mL of peptone water and homogenized for 30 s, producing the initial suspension (10^−1^ dilution). Serial decimal dilutions were prepared to obtain 10^−2^ and 10^−3^ dilutions. Analyses were performed immediately after “madalenas” preparation (T0), after three days (T3), and after six days (T6) to evaluate yeasts and molds, total viable mesophilic microorganisms, coliforms, and *Bacillus cereus*. Yeasts and molds were enumerated by surface-plating 0.2 mL aliquots onto pre-dried Dichloran Rose Bengal Chloramphenicol Agar (DRBC) followed by incubation at 25 °C for 5 days; yeast counts were determined on plates containing 15–300 colonies, while all mold colonies were recorded. Total viable mesophilic microorganisms were determined using Plate Count Agar (PCA) by the pour plate method, inoculating 1 mL of each dilution in duplicate, and incubating inverted at 30 °C for 72 h, with counts performed on plates containing 15–300 colonies. Coliforms were enumerated using Violet Red Bile Lactose Agar (VRBLA) under the same pour plate conditions, with incubation at 30 °C for 48 h and counting on plates with 10–150 colonies. *Bacillus cereus* was selectively enumerated by surface-plating 0.2 mL aliquots onto Mannitol Egg Yolk Polymyxin (MYP) agar and incubating at 37 °C for 24 h, with counts obtained from plates containing 15–300 colonies.

### 3.8. Statistical Analysis

The samples were analyzed in triplicate (*n* = 3), and the results were expressed as mean ± standard deviation. When the statistical analyses involved only two samples, a Student’s *t*-test was used to compare them. When dealing with three or more samples, analysis of variance (ANOVA) was applied; Tukey’s test was used to classify homoscedastic samples and Tahmane-T2 test was used for heteroscedastic samples.

For the analysis of the “madalenas” parameters, they were analyzed using a two-way ANOVA with type III sum of squares, in SPSS software (Version 25, IBM Corp, Armonk, NY, USA). This type of analysis allows treating the two factors storage time (ST) and formulation (F) independently, thereby enabling the assessment of the contribution of each factor irrespective of the other and providing a better understanding of the evolution of the parameters over storage. Throughout the work, the significance level was set at 0.05.

## 4. Conclusions

Citrus peel flours, particularly lime peel flour, are promising functional food ingredients due to their high dietary fiber, protein content, beneficial fatty acid profile, and rich bioactive composition. Lime peel flour exhibited the highest phenolic content and flavonoid predominance, correlating with superior antioxidant, antimicrobial, antiproliferative, and anti-inflammatory activities particularly hesperidin, limocitrin derivatives, and apigenin derivatives. Incorporation of lime peel flour into bakery products demonstrated its potential to enhance shelf life and maintain product quality while partially substituting wheat flour. This study highlights the valorization of citrus by-products, illustrating a circular economy approach that transforms agro-industrial waste into health-promoting food ingredients. Overall, lime peel flour represents a sustainable and nutritionally valuable ingredient, supporting the development of functional foods and contributing to environmental impact reduction in the food industry.

## Figures and Tables

**Table 1:** Nutritional composition (g/100 g in fresh weight) and energy value (kcal/100 g in fresh weight) of the flours produced.

Nutritional Components	FL	FT	FLA	FLO
Ash	0.54 ± 0.02 ^c^	0.60 ± 0.04 ^c^	1.18 ± 0.05 ^a^	0.8 ± 0.1 ^b^
Proteins	1.45 ± 0.03 ^b^	1.43 ± 0.04 ^b^	1.85 ± 0.02 ^a^	1.05 ± 0.03 ^c^
Fat	0.7 ± 0.1 ^a^	0.6 ± 0.1 ^a^	0.6 ± 0.1 ^a^	0.329 ± 0.004 ^b^
Carbohydrates	25.54 ± 0.03 ^a^	25.6 ± 0.1 ^a^	22.7 ± 0.2 ^b^	15.25 ± 0.05 ^c^
Fibers	11.8 ± 0.2 ^b^	11.8 ± 0.3 ^b^	15.6± 0.2 ^a^	6.9 ± 0.1 ^c^
Energy	114.12 ± 0.5 ^a^	113.7 ± 0.2 ^a^	104.1 ± 0.4 ^b^	69.2 ± 0.2 ^c^

Results are presented as mean ± standard deviation. Small letters indicate a significant difference where *p* < 0.05. Different letters show significant differences, while identical letters do not show significant differences. Orange flour (FL), tangerine flour (FT), lime flour (FLA), and lemon flour (FLO).

**Table 2:** Chemical composition of the studied citrus flour samples.

	FL	FT	FLA	FLO
**Free sugars (g/100 g fw)**				
Fructose	3.9 ± 0.1 ^a^	3.7 ± 0.1 ^a^	0.66 ± 0.01 ^c^	1.70 ± 0.02 ^b^
Glucose	3.9 ± 0.1 ^a^	2.700 ± 0.004 ^c^	1.590 ± 0.002 ^d^	1.90 ± 0.02 ^b^
Sucrose	1.66 ± 0.01 ^b^	2.9 ± 0.1 ^a^	0.70 ± 0.03 ^d^	0.65 ± 0.04 ^c^
Trehalose	0.17 ± 0.01 ^d^	0.3 ± 0.1 ^c^	0.30 ± 0.01 ^b^	0.2 ± 0.1 ^a^
Total	9.63 ± 0.04 ^a^	9.6 ± 0.1 ^a^	3.25 ± 0.04 ^c^	4.45 ± 0.02 ^b^
**Fatty acids (%)**				
C6:0	n.d.	n.d.	n.d.	0.84 ± 0.01
C8:0	0.260 ± 0.004	n.d.	n.d.	n.d.
C10:0	0.48 ± 0.02 ^a^	0.37 ± 0.01 ^b^	n.d.	0.47 ± 0.01 ^a^
C12:0	1.34 ± 0.01 ^b^	1.54 ± 0.01 ^a^	0.42 ± 0.01 ^d^	1.03 ± 0.03 ^c^
C13:0	n.d.	n.d.	n.d.	0.37 ± 0.01
C14:0	1.420 ± 0.001 ^c^	1.16 ± 0.04 ^d^	1.46 ± 0.02 ^b^	2.86 ± 0.01 ^a^
C15:0	n.d.	0.21 ± 0.01 ^c^	0.37 ± 0.01 ^b^	2.00 ± 0.01 ^a^
C16:0	15.6 ± 0.2 ^b^	15.5 ± 0.5 ^b^	13.9 ± 0.1 ^c^	22.3 ± 0.1 ^a^
C17:0	n.d.	n.d.	0.40 ± 0.01 ^b^	1.080 ± 0.001 ^a^
C18:0	3.28 ± 0.01 ^d^	3.70 ± 0.05 ^c^	4.14 ± 0.01 ^b^	3.80 ± 0.01 ^a^
C18:1n9c	16.8 ± 0.2 ^a^	16.1 ± 0.1 ^b^	13.1 ± 0.1 ^c^	7.20 ± 0.05 ^d^
C18:2n6c	48.3 ± 0.2 ^a^	50.5 ± 0.1 ^b^	47.3 ± 0.1 ^c^	32.48 ± 0.04 ^d^
C18:3n3	11.2 ± 0.2 ^b^	10.9 ± 0.4 ^b^	16.990 ± 0.002 ^a^	17.1 ± 0.1 ^a^
C20:0	n.d.	n.d.	n.d.	0.42 ± 0.01
C22:0	n.d.	n.d.	0.580 ± 0.002 ^b^	1.51 ± 0.04 ^a^
C22:1n9	n.d.	n.d.	n.d.	0.61 ± 0.01
C23:0	n.d.	n.d.	0.480 ± 0.003 ^b^	1.12 ± 0.01 ^a^
C24:0	1.30 ± 0.01 ^b^	n.d.	0.810 ± 0.004 ^c^	3.9 ± 0.1 ^a^
C24:1	n.d.	n.d.	n.d.	0.91 ± 0.02
SFA	23.7 ± 0.2 ^b^	22.5 ± 0.5 ^c^	22.6 ± 0.1 ^c^	41.7 ± 0.3 ^a^
MUFA	16.8 ± 0.2 ^a^	16.1 ± 0.1 ^b^	13.1 ± 0.1 ^c^	8.72 ± 0.03 ^d^
PUFA	59.50 ± 0.01 ^c^	61.4 ± 0.5 ^b^	64.3 ± 0.1 ^a^	49.58 ± 0.03 ^d^
**Organic Acids (mg/100 g fw)**				
Oxalic acid	0.123 ± 0.004 ^b^	0.134 ± 0.005 ^b^	0.15 ± 0.1 ^a^	0.106 ± 0.001 ^a^
Quinic acid	n.d.	n.d.	0.99 ± 0.04 ^a^	0.62 ± 0.02 ^b^
Malic acid	n.d.	0.29 ± 0.01 ^b^	0.162 ± 0.003 ^c^	0.20 ± 0.01 ^a^
Citric acid	1.6 ± 0.3 ^b^	4.63 ± 0.01 ^a^	n.d.	n.d.
Fumaric acid	n.d.	0.005 ± 0.001 ^a^	0.003 ± 0.001 ^b^	0.0026 ± 0.0004 ^b^
Ascorbic acid	0.021 ± 0.001 ^b^	0.01555 ± 0.00003 ^c^	0.004 ± 0.001 ^d^	0.030 ± 0.001 ^a^
Total	1.74 ± 0.3 ^b^	5.07 ± 0.02 ^a^	1.31 ± 0.03 ^c^	0.96 ± 0.04 ^c^

Results are presented as mean ± standard deviation. Small letters indicates a significant difference where *p* < 0.05. Caproic acid (C6:0); Caprylic acid (C8:0); Capric acid (C10:0); Lauric acid (C12:0); Tridecylic acid (C13:0); Myristic acid (C14:0); Pentadecanoic acid (C15:0); Palmitic acid (C16:0); Heptadecanoic acid (C17:0); Stearic acid (C18:0); Oleic acid (C18:1n9c) Linoleic acid (C18:2n6); Alpha-linolenic acid (C18:3n3); Arachidic acid (C20:0); Behenic acid (C22:0); Erucic acid (C 22:1n9); Tricosanoic acid (C23:0); Tetracosanoic acid (C24:0); Tetracosenoic acid (C24:1); SFA—Saturated fatty acids; MUFA—Monounsaturated fatty acids; PUFA—Polyunsaturated fatty acids; n.d.—not detected. Orange flour (FL), tangerine flour (FT), lime flour (FLA), and lemon flour (FLO).

**Table 3:** Retention time (Rt), wavelengths of maximum absorption in the visible region (λmax), mass spectrum data, identification and quantification of phenolic compounds present in flours obtained from orange peel (FL), tangerine peel (FT), lime peel (FLA) and lemon peel (FLO).

						Quantification (mg/g dw)			
Peak	Rt (min)	λmax (nm)	[M-H]^−^ (*m*/*z*)	MS^2^ (*m*/*z*)	Attempt at Identification	FL	FT	FLA	FLO
1	3.35	261	191	173 (100), 127 (21)	Quinic acid	1.94 ± 0.02	1.16 ± 0.02	1.23 ± 0.02	1.19 ± 0.03
2	3.89	295	315	169 (11), 152 (100)	Galoyl deoxyhexoside	2.29 ± 0.01	1.43 ± 0.03	1.24 ± 0.01	3.18 ± 0.03
3	4.01	321	353	191 (100), 179 (69), 161 (7), 135 (51)	3-*O*-caffeoylquinic acid	3.0 ± 0.03	1.93 ± 0.05	0.96 ± 0.01	2.26 ± 0.02
4	4.01	332	355	193 (100)	Ferulic acid hexoside isomer I	0.46 ± 0.02	1.26 ± 0.03	0.37 ± 0.003	0.7 ± 0.01
5	4.52	310	355	193 (100)	Ferulic acid hexoside isomer II	nd	nd	0.53 ± 0.03	nd
6	4.72	311	385	223 (100), 179 (3)	Sinapic acid hexose	nd	nd	0.327 ± 0.004	nd
7	4.83	313	355	223 (21), 205 (11)	Sinapic acid pentoside	nd	nd	0.336 ± 0.002	nd
8	4.94	334	355	193 (100), 175 (15), 161 (6)	Ferulic acid hexoside isomer III	0.282 ± 0.002	nd	0.50 ± 0.01	nd
9	5.08	318	337	191 (31), 163 (100), 155 (3)	*p*-Coumaroylquinic acid	nd	nd	0.47 ± 0.02	nd
10	5.53	332	593	505 (14), 473 (24), 383 (18), 353 (29), 325 (11)	Apigenin-6,8-*C*-diglucoside	1.32 ± 0.01	0.86 ± 0.03	5.0 ± 0.2	0.65 ± 0.03
11	6.21	351	623	503 (45), 383 (100)	Diosmestin-6,8-di-*C*-glucoside	1.86 ± 0.03	0.71 ± 0.02	1.56 ± 0.02	0.90 ± 0.03
12	6.96	345	623	605 (3), 533 (13), 503 (100), 413 (21), 384 (45)	Quercetin-6,8-di-*C*-glucoside	1.99 ± 0.03	0.192 ± 0.002	2.95 ± 0.02	0.245 ± 0.001
13	7.31	318	739	431 (100)	Rhoifoline glucoside	0.175 ± 0.002	0.197 ± 0.002	nd	0.203 ± 0.004
14	8.13	825	625	299 (100)	Dihexosyldiosmetin	0.218 ± 0.002	0.193 ± 0.001	nd	0.75 ± 0.03
15	8.43	339	577	293 (100), 413 (44)	Vitexin rhamnoside	0.246 ± 0.002	nd	nd	nd
16	9.56	332	651	633 (100), 427 (51), 265 (22), 103 (10)	Sinapic acid benzoyl dihexoside	1.22 ± 0.02	0.175 ± 0.002	3.34 ± 0.01	0.186 ± 0.001
17	11.81	336	563	443 (30), 413 (100), 341 (28), 313 (15), 293 (48)	Apigenin-*O*-pentosyl-6-*C*-hexoside	0.437 ± 0.002	0.338 ± 0.002	0.93 ± 0.01	0.360 ± 0.003
18	12.21	324	595	287 (100)	Eriocitrin	0.61 ± 0.03	0.225 ± 0.004	1.17 ± 0.02	0.222 ± 0.002
19	12.58	336	595	287 (100)	Eriodictyol-*O*-neosperidoside	nd	nd	0.8 ± 0.03	nd
20	12.81	351	609	301 (100)	Quercetin3-*O*-rutinoside	0.424 ± 0.003	0.151 ± 0.002	1.20 ± 0.04	0.57 ± 0.02
21	13.17	345	593	285 (100)	Isosakuranetin-rutinoside	1.40 ± 0.04	0.365 ± 0.003	2.09 ± 0.02	0.418 ± 0.002
22	13.51	331	593	285 (100)	Dihydroxy-methoxy-flavanone rutinoside	nd	nd	nd	0.203 ± 0.004
23	14.01	321	649	605 (55), 433 (100), 269 (22)	Malonylated Genistein-7-*O*-xylosylglucoside	nd	nd	nd	0.229 ± 0.004
24	14.48	324	593	285 (100)	Luteolin rutinoside	0.361 ± 0.001	0.36 ± 0.002	nd	0.60 ± 0.03
25	14.73	334	461	341 (100), 299 (21)	Diosmestin-*C*-hexoside	0.64 ± 0.02	nd	nd	nd
26	15.29	339	461	299 (100)	Diosmestin-8-*C*-glucoside	1.19 ± 0.03	0.200 ± 0.001	2.09 ± 0.02	0.171 ± 0.003
27	15.91	329	579	271 (100)	Narirutin	nd	0.204 ± 0.002	2.36 ± 0.02	0.274 ± 0.002
28	16.43	341	623	315 (100)	Isorhamnetin-3-*O*-rutinoside	1.27 ± 0.02	0.246 ± 0.004	3.61 ± 0.21	0.223 ± 0.004
29	17.34	341	607	503 (16), 383 (35), 299 (100)	Neodiosmin	0.55 ± 0.02	0.317 ± 0.002	nd	0.199 ± 0.002
30	18.21	334	609	301 (100)	Hesperidin	1.9 ± 0.03	0.76 ± 0.04	23.5 ± 0,2	0.48 ± 0.02
31	18.51	345	681	373 (100), 211 (21)	Limocitrol-3-*O*-hexoside-7-*O*-rutinoside	nd	nd	1.363 ± 0.005	nd
32	19.43	358	651	489 (28), 651 (13), 345 (100)	Limocitrin-glucosyl-3-hydroxy-3-methylglutaryl-glucoside	nd	nd	6.42 ± 0.23	nd
33	19.65	352	681	373 (100), 211 (21)	Limocitrol-*O*-hexoside-*O*-deoxyhexoside	2.06 ± 0.03	0.144 ± 0.002	13.2 ± 0.2	0.209 ± 0.002
34	21.48	315	351	163 (100)	Cinnamic acid-3-*O*-acetylhexoside	0.285 ± 0.003	nd	2.02 ± 0.02	nd
35	22.69	338	795	693 (18), 651 (33), 345 (100)	Limocitrin-*O*-glucoside-di(hydroxyl-3-methylglutaryl) isomer I	0.262 ± 0.004	nd	1.36 ± 0.01	nd
36	23.47	353	795	693 (18), 651 (33), 345 (100)	Limocitrin-*O*-glucoside-di(hydroxyl-3-methylglutaryl) isomer II	0.61 ± 0.02	nd	3.9 ± 0.3	nd
37	27.61	329	593	285 (100)	Luteolin-neohesperidoside	nd	0.211 ± 0.003	nd	0.276 ± 0.002
38	29.04	323	751	547 (100), 505 (33), 463 (20), 301 (3)	Quercetin-acetylhexoside-malonylhexose	nd	nd	2.65 ± 0.01	nd
39	36.99	327	487	355 (16), 337 (26), 217 (18), 193 (100), 149 (19)	Ferulic acid-*O*-pentosylhexoside	nd	nd	2.26 ± 0.02	nd
40	38.11	nd	471	453 (100), 325 (33)	Diacetylnomylin	nd	0.213 ± 0.001	1.19 ± 0.02	0.325 ± 0.002
41	38.53	312	325	163 (100)	*p*-Coumaric acid-*O*-glucoside isomer I	nd	nd	1.2 ± 0.03	nd
42	39.42	nd	513	495 (58), 469 (33), 325 (100)	Nomilin	nd	0.162 ± 0.003	nd	nd
43	40.46	nd	469	325 (100)	Limonin	nd	0.64 ± 0.03	nd	0.219 ± 0.004
44	41.88	334	297	265 (63), 175 (100), 113 (18)	Benzol glucuronide	nd	0.315 ± 0.001	nd	0.227 ± 0.003
45	43.15	334	325	163 (100)	*p*-Coumaric acid-*O*-glucoside isomer II	nd	0.93 ± 0.03	nd	2.09 ± 0.01
46	44.57	341	311	147 (100)	Cinnamyl alcohol glucoside	nd	0.64 ± 0.02	nd	0.295 ± 0.004
					Total phenolic acids	9.48 ± 0.1	7.8 ± 0.2	14.8 ± 0.2	10.1 ± 0.1
					Total flavonoids	17.5 ± 0.3	4.8 ± 0.1	76 ± 2	7.2 ± 0.2
					Total phenolic compounds	27.0 ± 0.4	13.5 ± 0.3	91 ± 2	17.3 ± 0.3

nd—not detected. Quantification results are presented as mean ± standard deviation. Orange flour (FL), tangerine flour (FT), lime flour (FLA), and lemon flour (FLO).

**Table 4:** Bioactive potential of citrus flours.

				**FL**	**FT**	**FLA**	**FLO**
**Antioxidant activity (EC_50_ values, μg/mL)**							
**TBARS**				0.83 ± 0.04 ^a^	0.86 ± 0.04 ^a^	0.29 ± 0.04 ^b^	0.9 ± 0.3 ^a^
**DPPH**				5.4 ± 0.4 ^a^	5 ± 1 ^a^	2.01 ± 0.03 ^b^	2.0 ± 0.3 ^b^
**Reduction Power**				0.89 ± 0.04 ^d^	3.54 ± 0.05 ^b^	2.9 ± 0.1 ^c^	4.3 ± 0.1 ^a^
**Antiproliferative activity (GI_50_ values, μg/mL)**							
Tumor cell line		AGS		>400	>400	>400	>400
		Caco-2		>400	>400	140 ± 3	>400
		MCF-7		>400	>400	343 ± 15	>400
		NCl-H460		>400	>400	208 ± 12	>400
Non-tumor cell line		PLP2		>400	>400	385 ± 10	>400
**Nitric oxide (NO)-production inhibition (IC_50_ values, μg/mL)**							
RAW 264.7				>400	>400	>400	>400
**Antimicrobial Activity (mg/mL)**							
**Bacteria**		*Staphylococcus* *aureus*	*Bacillus cereus*	*Listeria monocytogenes*	*Escherichia coli*	*Salmonella* Typhimurium	*Enterobacter cloacae*
**FL**	MIC	2	0.5	1	1	1	2
	MBC	4	1	2	2	2	4
**FT**	MIC	2	1	1	2	1	1
	MBC	4	2	2	4	2	2
**FLA**	MIC	1	1	1	1	1	1
	MBC	2	2	2	2	2	2
**FLO**	MIC	2	1	1	1	1	2
	MBC	4	2	2	2	2	4
**E211**	MIC	4.0	0.5	1.0	1.0	1.0	2.0
	MBC	4.0	0.5	2.0	2.0	2.0	4.0
**E224**	MIC	1.0	2.0	0.5	0.5	1.0	0.5
	MBC	1.0	4.0	1.0	1.0	1.0	0.5
**Fungi**		*Aspergillus* *fumigatus*	*Aspergillus* *niger*	*Aspergillus versicolor*	*Penicillium funiculosum*	*P. verrucosum var. cyclopium*	*Trichodermerma viride*
**FL**	MIC	0.25	0.5	1	0.5	1	0.25
	MBC	0.5	1	2	1	2	0.5
**FT**	MIC	0.25	0.125	1	0.25	1	0.5
	MBC	0.5	0.25	2	0.5	2	1
**FLA**	MIC	0.25	0.5	1	0.25	0.5	0.5
	MBC	0.5	1	2	0.5	1	1
**FLO**	MIC	0.25	0.25	0.5	0.25	1	0.25
	MBC	0.5	0.5	1	0.5	2	0.5
**E211**	MIC	1.0	1.0	2.0	1.0	2.0	1.0
	MBC	2.0	2.0	2.0	2.0	4.0	2.0
**E224**	MIC	1.0	1.0	1.0	0.5	1.0	0.5
	MBC	1.0	1.0	1.0	0.5	1.0	0.5

Results are presented as mean ± standard deviation. Small letters indicates a significant difference (*p* < 0.05). Different letters show significant differences, while identical letters do not show significant differences. Controls: sodium sulfite (E211) and potassium metabisulfite (E224), Ellipticine: 1.23 ± 0.03 µg/mL (AGS), 1.21 ± 0.02 µg/mL (Caco-2), 1.02 ± 0.02 µg/mL (MCF-7), 1.03 ± 0.09 (NCI-H460), 1.4 ± 0.1 µg/mL (PLP2). IC_50_ values for Dexamethasone: 6.3 ± 0.4 µg/mL (RAW 264.7). MIC—Minimum Inhibitory Concentration, MBC—Minimum Bactericidal Concentration, MFC—Minimum Fungicidal Concentration. Orange flour (FL), tangerine flour (FT), lime flour (FLA), and lemon flour (FLO).

**Table 5:** Colorimetric, water activity and texture parameters in wheat-based madeleines, with synthetic preservative and with the addition of citrus extract at different concentrations, evaluating storage time (ST) and formulation (F).

**Colour Parameters**								
		**External**			**Internal**			
		L*	a*	b*	L*	a*	b*	
**Storage time (ST)**	0 days	55 ± 5 ^a^	11 ± 2	41 ± 2	66 ± 6 ^a^	−2 ± 1 ^a^	38 ± 6 ^a^	
	3 days	58 ± 6 ^b^	11 ± 1	42 ± 2	70 ± 6 ^b^	−1 ± 1 ^b^	43 ± 7 ^b^	
	6 days	59 ± 6 ^b^	11 ± 3	42 ± 2	68 ± 6 ^b^	−2 ± 1 ^a^	39 ± 6 ^a^	
***p*-value (*n* = 12)**	ANOVA	<0.001	0.787	0.726	<0.001	0.001	<0.001	
**Formulation (F)**	MFT	63 ± 2 ^c^	8 ± 2	43 ± 2 ^b^	73 ± 2 ^b^	−2 ± 0.3 ^a^	34 ± 2 ^a^	
	MCS	62 ± 2 ^c^	12 ± 1	42 ± 1 ^a,b^	74 ± 3 ^b^	−2.1 ± 0.3 ^a^	33 ± 2 ^a^	
	M10	54 ± 2 ^b^	12 ± 2	42 ± 1 ^a,b^	64 ± 2 ^a^	−1.3 ± 0.4 ^b^	45 ± 2 ^b^	
	M20	51 ± 3 ^a^	12 ± 2	39 ± 3 ^a^	61 ± 3 ^a^	−1 ± 1 ^c^	47 ± 4 ^b^	
***p*-value (*n* = 9)**	ANOVA	<0.001	<0.001	0.011	<0.001	<0.001	<0.001	
**ST × F (*n* = 36)**	*p*-value	0.902	0.033	0.841	0.785	0.158	0.111	
**Water activity and texture parameters**								
		aw	Hardness (g)	Elasticity (%)	Cohesiveness (%)	Viscosity (%)	Chewing (%)	Resilience (%)
**Storage time (ST)**	0 days	0.90 ± 0.01 ^b^	1033 ± 271	0.93 ± 0.02	0.74 ± 0.02	770 ± 173	733 ± 150	0.38 ± 0.01
	3 days	0.90 ± 0.01 ^b^	1320 ± 282	0.92 ± 0.02	0.68 ± 0.04	870 ± 176	813 ± 164	0.32 ± 0.02
	6 days	0.89 ± 0.01 ^a^	1477 ± 358	0.92 ± 0.03	0.7 ± 0.1	978 ± 178	901 ± 174	0.31 ± 0.03
***p*-value (*n* = 12)**	ANOVA	0.002	<0.001	0.605	<0.001	<0.001	<0.001	<0.001
**Formulation (F)**	MFT	0.90 ± 0.01	1085 ± 202	0.93 ± 0.02 ^a^	0.72 ± 0.03	763 ± 136	737 ± 91	0.35 ± 0.03
	MCS	0.90 ± 0.01	926 ± 159	0.91 ± 0.01 ^a^	0.72 ± 0.02	666 ± 74	608 ± 62	0.34 ± 0.02
	M10	0.90 ± 0.01	1450 ± 227	0.93 ± 0.01 ^a^	0.70 ± 0.04	1015 ± 103	932 ± 84	0.35 ± 0.04
	M20	0.90 ± 0.01	1644 ± 237	0.93 ± 0.03 ^a^	0.64 ± 0.05	1047 ± 80	985 ± 90	0.31 ± 0.04
***p*-value (*n* = 9)**	ANOVA	0.594	<0.001	<0.001	<0.001	<0.001	<0.001	<0.001
**ST × F (*n* = 36)**	*p*-value	0.163	0.001	0.153	<0.001	<0.001	0.021	<0.001

The standard deviations presented were calculated from results obtained under different operating conditions. Therefore, these values should not be considered a measure of precision, but rather the range of recorded values. Small letters indicate a significant difference where *p* < 0.05. Different letters show significant differences, while identical letters do not show significant differences. L* luminosity; a* colour axis from green (−) to red (+); b* colour axis from blue (−) to yellow (+). Orange flour (FL), tangerine flour (FT), lime flour (FLA), and lemon flour (FLO).

## Data Availability

The original contributions presented in this study are included in the article. Further inquiries can be directed to the corresponding author.
